# Effects of Sevoflurane and Propofol on Posttraumatic Stress Disorder After Emergency Trauma: A Double-Blind Randomized Controlled Trial

**DOI:** 10.3389/fpsyt.2022.853795

**Published:** 2022-02-25

**Authors:** Junfeng Zhong, Yan Li, Lichao Fang, Dan Han, Chuhao Gong, Shuangyan Hu, Rongguo Wang, Liwei Wang, Rui Yao, Beiping Li, Yangzi Zhu, Youjia Yu

**Affiliations:** ^1^Department of Pain, Shaoxing People's Hospital, Shaoxing, China; ^2^Department of Anesthesiology, Suzhou Xiangcheng People's Hospital, Suzhou, China; ^3^Emergency and Critical Department, Suzhou Xiangcheng People's Hospital, Suzhou, China; ^4^Department of Anesthesiology, Xuzhou Renci Hospital, Xuzhou, China; ^5^Department of Anesthesiology, Shaoxing People's Hospital, Shaoxing, China; ^6^Department of Anesthesiology, Xuzhou Central Hospital, Xuzhou, China; ^7^Department of Anesthesiology, The First People's Hospital of Xuzhou, Xuzhou, China

**Keywords:** trauma, posttraumatic stress disorder, propofol, sevoflurane, emergency surgery

## Abstract

**Objective:**

Posttraumatic stress disorder (PTSD) is a frequent and disabling consequence of traumatic events. A previous study found that early use of propofol was a potential risk factor for PTSD. This prospective study aimed to investigate the effect of propofol and sevoflurane on PTSD after emergency surgery in trauma patients.

**Methods:**

A total of 300 trauma patients undergoing emergency surgery were randomly divided into two groups and anesthetized with propofol and/or sevoflurane. Perioperative clinical data were collected. The incidence of PTSD was evaluated with the Clinician-Administered PTSD Scale for DSM-5 (CAPS-5) in the two groups 1 month after the operation. The relevance of the injury time and CAPS-5 scores was assessed by Spearman correlation analysis. Logistic regression analysis was used to analyze the risk factors for PTSD.

**Results:**

The incidence of PTSD in the propofol group was higher than that in the sevoflurane group 1 month postoperatively (23.2 vs. 12.2%, *P* = 0.014). The injury time was negatively correlated with the CAPS-5 score in the propofol group (*r* = *-*0.226*, P* < 0.001). In the logistic regression analysis, the utilization of propofol was an independent risk factor for PTSD (*P* = 0.017).

**Conclusion:**

Early use of propofol general anesthesia in emergency surgery for trauma patients may increase the risk of PTSD.

**Clinical Trial Registration:**

www.chictr.org.cn, identifier: ChiCTR2100050202.

## Introduction

Posttraumatic stress disorder (PTSD) refers to a set of characteristic symptoms that occurs after an individual experiences an unusually threatening or catastrophic event: repeated recurrence of traumatic experiences, persistent increased alertness, and avoidance of situations similar to or related to stimuli that may persist for years or decades in trauma-exposed survivors (1–3). As a result, this mental state is highly debilitating and interferes with the patient's daily life and social activities (4, 5). In recent years, due to frequent traffic accidents, natural disasters, wars, terrorist violence and other events, the incidence of PTSD has been as high as 10–22% (6, 7). It has caused a large amount of harm and placed a large economic burden on patients, families and society (8). However, until now, prevention and treatment options for PTSD have been limited (9).

Patients who experience physical trauma in traumatic events such as car accidents, earthquakes and falls often need emergency surgical treatment or intensive care at an early stage. Studies have identified the prevalence of PTSD in intensive care unit (ICU) survivors to be more than twice that in the general population (10). Approximately 20% of patients develop PTSD after surgery or hospitalization (11). These trauma patients are inevitably treated with anesthetic sedatives that act on the central nervous system, propofol being the most commonly used (12). Once PTSD develops, it is difficult to cure (13). An important reason for this is that conditioned fear memory is abnormally strengthened and does not easily fade away (14, 15). One animal study suggested that early application of propofol has a significantly strengthens fear memory (16). A clinical retrospective analysis reported that early use of propofol in ICU patients who experienced car accident trauma was a risk factor for PTSD (17). However, there is no prospective clinical study on the effect of the use of different general anesthetics on PTSD in emergency trauma patients.

The purpose of this study was to investigate the effects of propofol and sevoflurane, two commonly used general anesthetics, on the incidence of postoperative PTSD in emergency trauma patients to lay a theoretical foundation for finding a more suitable general anesthetic for emergency trauma patients.

## Materials and Methods

### Study Design and Participants

This study was registered at www.chictr.org.cn (ChiCTR2100050202). The study protocol was approved by the ethics committees of all participating hospitals. Written informed consent was obtained from all patients. Three hundred trauma patients who underwent emergency surgery under general anesthesia were selected. The inclusion criteria were as follows: emergency trauma due to a car accident, falling, engineering accident, etc., ASA I-III, and age 18–60. The exclusion criteria were craniocerebral or spinal cord injury, hemorrhagic shock decompensation, liver or renal dysfunction, history of alcohol abuse or drug dependence, history of neurological or psychiatric diseases, severe visual, hearing or language impairment, and significant past physical or mental trauma.

### Anesthesia-Related Procedures

Patients were randomly divided into the propofol group and the sevoflurane group by the random number method. Anesthesia induction and endotracheal intubation were performed after admission to the operating room. Anesthesia induction was performed with midazolam (20–60 μg/kg), sufentanil (0.4–0.8 μg/kg), etomidate (0.3 mg/kg) and cisatracurium (0.2–0.4 mg/kg). After successful tracheal intubation and mechanical ventilation were achieved, the tidal volume was 6–10 mL/kg, and the respiratory rate was 11–14 times/min. The propofol group received 1% propofol at 0.1–0.15 mg·kg^−1^·min^−1^ for anesthesia maintenance. The sevoflurane group received 2–4% sevoflurane via inhalation for anesthesia maintenance. All patients received remifentanil continuously at 0.2~0.4 μg·kg-1·min-1 during surgery to maintain analgesia, and cisatracurium was intermittently injected intravenously to maintain muscle relaxation. BP, ECG, HR, SpO_2_, PetCO_2_ and the Bispectral Index (BIS) were continuously monitored intraoperatively. The intraoperative SpO_2_ was maintained above 98%, and the rate of propofol or sevoflurane inhalation was adjusted according to the BIS to maintain the BIS between 40 and 60 and the PetCO_2_ between 35 and 45 mmHg. Intravenous analgesia was used in all patients after surgery. The analgesic fluid formulations in both groups were as follows: sufentanil combined with palonosetron hydrochloride (0.15 mg diluted to 200 ml. Sufentanil patient-controlled analgesia was infused at a rate of 0.04 μg·kg^−1^·h^−1^ and a 0.1 μg bolus every 15 min when needed *via* the pump for the first 2 days after surgery.

### Observational Index

General demographic data were collected preoperatively, and patients were assessed with the Acute Physiology and Chronic Health Evaluation II (APACHE II) score and trauma severity score (TSS). The patient's clinical parameters, such as injury time (the time from injury to anesthesia induction), drug use, operation time, blood loss, transfusion and ICU admission, were recorded. Adverse reactions, such as pain, delirium, nausea, and pruritus, were observed 48 h after surgery, and visual analog scale (VAS) scores were recorded 6, 24, and 48 h after surgery. The occurrence of PTSD was assessed with the Clinician-Administered PTSD Scale for DSM-5 (CAPS-5) 1 month after surgery. Professionally trained nurses, blinded to treatment group assignments, carried out the neuropsychological tests at both times in tranquil surroundings. The data analyst also did not know the grouping. The CAPS-5 is a structured diagnostic interview and considered the gold standard in PTSD evaluation. The CAPS-5 provides a continuous measure of the severity of overall PTSD and of the four symptom clusters (intrusions, avoidance, negative alterations in cognition/mood, arousal and reactivity) and presence/absence of PTSD diagnosis, which can be administered by appropriately trained paraprofessionals (18).

### Statistics

PASS software was used to estimate the sample size. According to pretrial results, the prevalence of PTSD was 9% in the sevoflurane group and 23% in the propofol group. Hence, to detect a reduction in the PTSD rate from 23 to 9% and achieve a statistical efficacy of 90% (α = 0.05, β = 0.1), 278 patients would be required.

SPSS 23.0 software was used for the statistical analysis. The data are expressed as the mean ± standard deviation, the independent sample *t* test was used for comparison of the means of the two groups, and the *t* test was used when the variance was not uniform. Enumeration data were compared by χ*2* test. A logistic regression model was established to analyze the risk factors related to PTSD in emergency trauma patients. The Hosmer-Lemeshow test was used. When *P* < 0.05, the difference was statistically significant. Spearman's test was used to analyze the correlation between the CAPS-5 score and the time to anesthesia, i.e., the time from injury to the start of anesthesia; when the test level was α = 0.05, *P* < 0.05 was considered statistically significant.

## Results

### Demographic Information and Perioperative Clinical Data

**Figure 1:**
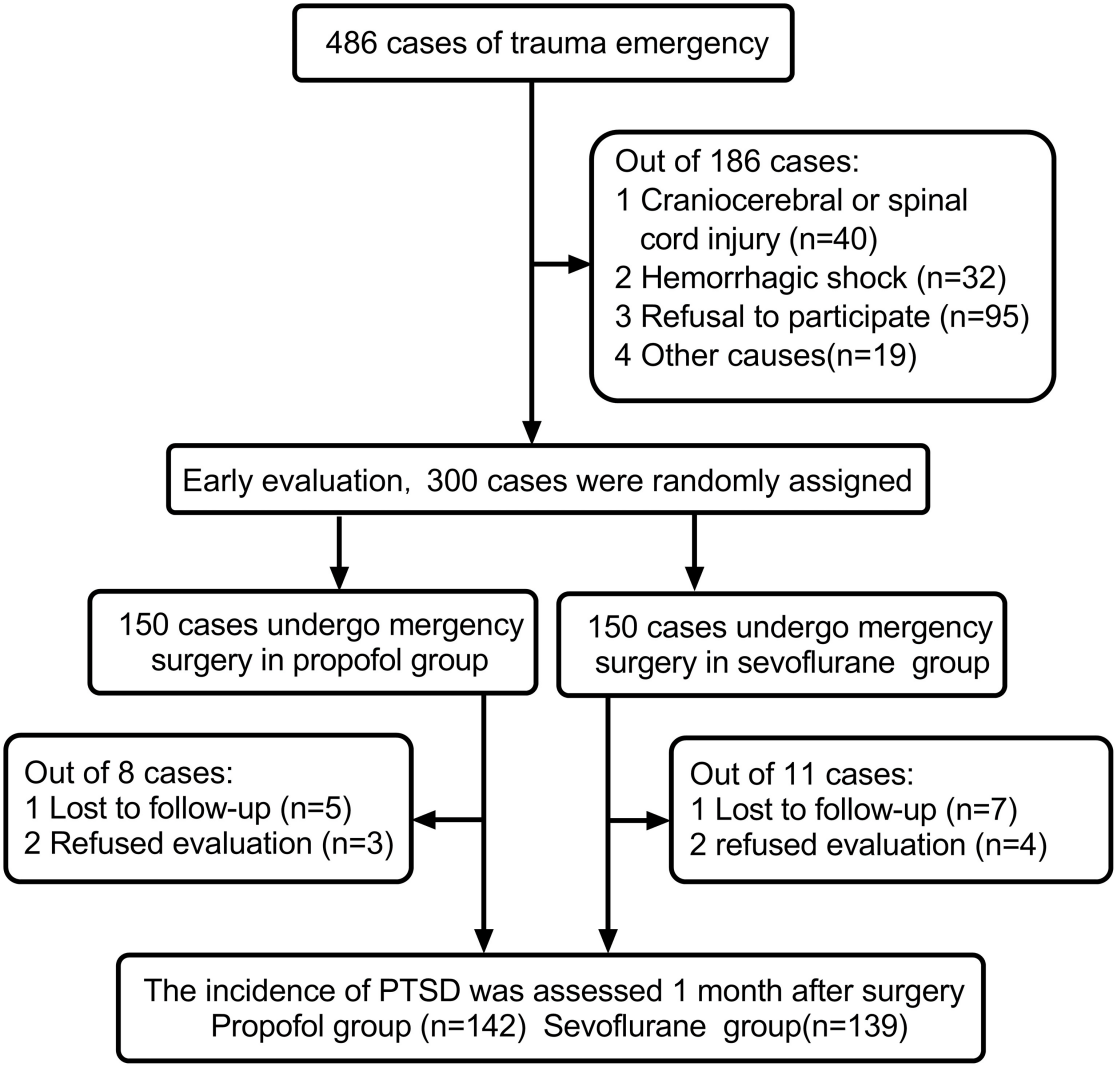
Enrollment flowchart for patients in this study.

A total of 300 patients were enrolled. Nineteen patients withdrew from the study due to refusal to return for follow-up, loss of follow-up and other reasons. Finally, 281 patients completed the follow-up and cognitive function assessments. The process is shown in Figure 1. There were no statistically significant differences in sex distribution, age, ASA grade, preoperative hypertension, smoking status, APACHE II score, ISS, injury time, operation, recovery time, blood loss, transfusion or ICU occupancy rate between the two groups, as shown in Table 1. Postoperative pain, delirium, nausea and pruritus between the two groups showed no statistical significance, as shown in Table 2.

**Table 1:** Demographic and surgery characteristics.

**Factor**	**Propofol group** **(*n* = 142)**	**Sevoflurane group** **(*n* = 139)**	**t/*χ^2^***	***P*-value**
Age (years)	40.3 ± 9.3	41.1 ± 8.7	0.776	0.438
BMI (kg/m^2^)	27.7 ± 5.2	27.1 ± 4.8	0.932	0.352
Sex (male/female)	82/60	77/62	0.158	0.691
Hypertension (cases, %)	17 (12%)	14 (10.1%)	0.258	0.611
Diabetes (cases, %)	15 (10.6%)	16 (11.5%)	0.064	0.800
Smoking (cases, %)	27 (19%)	25 (18%)	0.049	0.824
ASA grade (II/III)	98/44	100/39	0.289	0.591
APACHE II score	7.68 ± 2.49	7.54 ± 2.70	0.463	0.643
ISS	14.3 ± 8.11	13.5 ± 8.32	0.786	0.433
Injury time (min)	53.3 ± 9.78	52.7 ± 8.19	0.528	0.598
Surgery time (min)	103 ± 40.7	99 ± 39.5	0.827	0.409
Awake time (min)	19.6 ± 9.30	20.2 ± 8.31	0.599	0.549
Intraoperative awareness (cases, %)	0	0		
**ICU admission (cases, %)**	16(11.3%)	11(7.9%)	0.910	0.340
**Estimated blood loss (ml)**	382 ± 210	414 ± 222	−1.243	0.215
**Transfusion (cases, %)**	10(7.0%)	12(8.6%)	0.246	0.620

*Physiology and Chronic Health Evaluation; ISS, trauma severity score. Values are the mean ± SD or number (percentage)*.

**Table 2:** Adverse characteristics in the two groups.

**Factor**	**Propofol group** **(*n* = 142)**	**Sevoflurane group** **(*n* = 139)**	**t/*χ^2^***	***P*-value**
VAS score 6 h postoperation	3.1± 1.3	2.9 ± 1.4	0.930	0.353
VAS score 24 h postoperation	3.2 ± 1.6	3.1 ± 1.4	0.629	0.530
VAS score 48 h postoperation	1.8 ± 1.5	1.7 ± 1.4	0.794	0.428
Delirium (cases, %)	19 (13.4%)	17 (12.2%)	0.083	0.773
Nausea (cases, %)	21 (14.8%)	20 (14.4%)	0.009	0.924
Pruritus (cases, %)	13 (9.2%)	14 (10.1%)	0.068	0.794

*VAS, visual analog scale for pain. Values are the mean ± SD or number (percentage)*.

### Effects of General Anesthetic on PTSD

Comparison of the incidence of PTSD between the two groups showed that PTSD occurred in 33 of 142 patients in the propofol group (23.2%). PTSD occurred in 17 of 139 patients in the sevoflurane group (12.2%). Comparing the CAPS-5 scores between the two groups, the CAPS-5 score in the propofol group was significantly higher than that in the sevoflurane group, as shown in Table 3.

**Table 3:** CAPS-5 scores and the incidence of adverse events in the two groups.

**Factor**	**Propofol group** **(*n* = 142)**	**Sevoflurane group** **(*n* = 139)**	**t/*χ^2^***	***P*-value**
CAPS-5 score	34.1 ± 5.8	32.2 ± 6.8	2.46	0.014
PTSD (cases, %)	33 (23.2%)	17 (12.2%)	5.82	0.016

*CAPS-5, Clinician-Administered PTSD Scale; PTSD, posttraumatic stress disorder. Values are the mean ± SD or number (percentage)*.

### Correlation Analysis for the Time to Anesthesia

**Figure 2:**
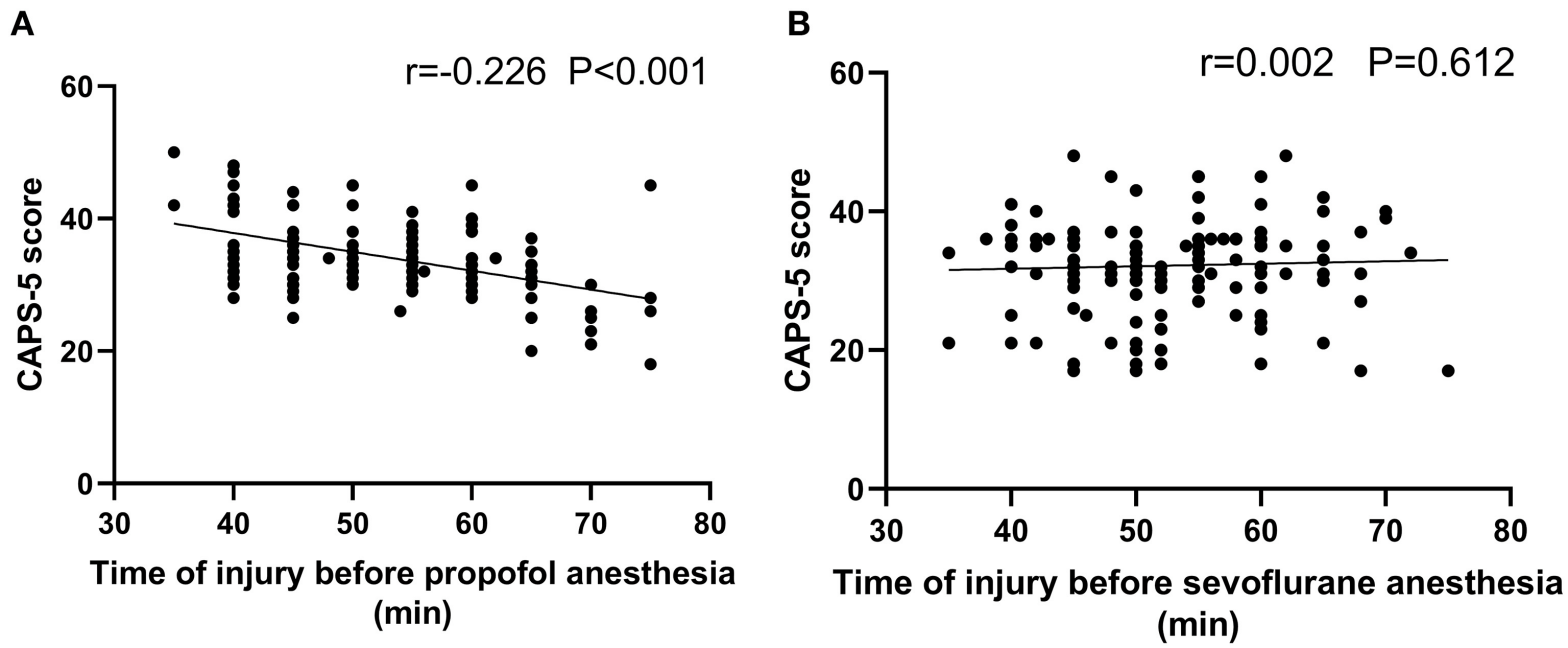
Correlation analysis for the time from injury to admission and the CAPS-5 score 1 month after surgery. **(A)** shows the propofol general anesthesia group (*n* = 142), and **(B)** shows the sevoflurane general anesthesia group (*n* = 139). Scatter plot **(A)** shows a significant negative correlation between the time from injury to admission and the CAPS-5 score 1 month after surgery (*r* = −0.226; *p* < 0.001), and **(B)** shows no significant correlation between the time from injury to admission and the CAPS-5 score 1 month after surgery (*r* = 0.002; *p* = 0.612).

Spearman analysis was performed on the correlation between the time to anesthesia and the CAPS-5 score in the two groups. The results showed that the time to anesthesia was negatively correlated with the CAPS-5 score in the propofol group (*r* = 0.226, *P* < 0.001). There was no significant negative correlation between the time to anesthesia and the CAPS-5 score in the sevoflurane group (*r* = 0.002, *P* = 0.612), as shown in Figure 2.

### Risk Factors for PTSD in Emergency Trauma Patients

Risk factors associated with PTSD in emergency trauma patients were assessed by logistic regression analysis. Sex, age, operation time, trauma severity and other factors were not significantly correlated with the incidence of PTSD, but the use of propofol was significantly correlated with the incidence of PTSD (*P* = 0.017), as shown in Table 4.

**Table 4:** Logistic regression analysis for factors related to postoperative PTSD in patients with emergency trauma.

**Factor**	**OR**	**95% credibility interval**	***P*-value**
Gender	0.711	0.342~1.475	0.359
BMI	0.984	0.921~1.051	0.625
Age	0.997	0.962~1.033	0.857
Hypertension	1.336	0.450~3.970	0.602
Diabetes	0.683	0.263~1.776	0.435
Smoking	0.516	0.220~1.210	0.128
Injury time	0.968	0.934~1.004	0.083
Delirium	1.007	0.382~2.655	0.989
Postoperation 6 h VAS	0.914	0.714~1.169	0.473
Postoperation 24 h VAS	0.966	0.777~1.202	0.759
Postoperation 48 h VAS	0.842	0.656~1.081	0.176
Anesthetics (propofol)	2.236	1.152~4.340	0.017
ICU admission	0.409	0.162~1.032	0.058

## Discussion

Epidemiological studies have shown that the prevalence of PTSD in the general U.S. population is 6–8%, and it can be as high as 13–30% in the military (17, 19). More than 230 million patients worldwide undergo surgery each year, and 20% of patients develop PTSD after surgery or hospitalization (11, 20). In this study, among 281 patients who underwent emergency trauma surgery, 50 (17.8%) developed PTSD 1 month after surgery. The incidence of PTSD in the propofol anesthesia group was significantly higher than that in the sevoflurane anesthesia group. The above results show that the incidence of PTSD in emergency trauma patients is not low and that the occurrence of PTSD is affected by early anesthesia treatment.

The pathogenesis of PTSD is complex, and its exact neurobiological mechanism is still unclear (21). Recurrent traumatic experiences, one of the core symptoms of PTSD, have been found to be associated with abnormal enhancement of fear memory (22, 23). Anesthetics are commonly thought to have an amnestic effect (24, 25), but the most recent studies found not only that propofol was relatively ineffective at inhibiting activation of the amygdala-dependent fear system but also that it had an unusual strengthening effect on emotional memories (26, 27). In an animal experiment, it was found that propofol anesthesia could significantly enhance fear memory in experimental animals within 1 h after receiving the conditioned fear stimulus. However, after 1 h, propofol had no significant effect on fear memory, and it was thought that propofol might enhance memory traces in the early consolidation stage of fear memory (16). To this end, this study independently analyzed the correlation between the time to anesthesia and the CAPS-5 score 1 month after propofol and sevoflurane anesthesia. The results showed that the time to anesthesia was negatively correlated with the CAPS-5 score in the propofol group, while there was not a significant negative correlation between the time to anesthesia and the CAPS-5 score in the sevoflurane group. This finding is consistent with animal experiments, suggesting that earlier application of propofol in the early stage of trauma may enhance the degree of traumatic memory reinforcement and thus promote the occurrence of PTSD.

PTSD may be associated with a variety of factors, such as sex, pain, trauma severity, and smoking (28, 29). To identify additional related risk factors, logistic regression analysis was performed on all participants to evaluate the risk factors related to PTSD in emergency trauma patients. Sex, age, operation time, trauma severity and other factors were not significantly correlated with the incidence of PTSD, but the use of propofol was significantly correlated with the incidence of PTSD. These results provide further evidence that propofol use is associated with an increased risk of PTSD.

Nevertheless, this study had several limitations that deserve mention. First, the 6.3% of patients lost by the 1-month follow-up may have resulted in statistical limitations of the PTSD incidence at the 1-month follow-up. Second, surgery itself may have an effect on PTSD. However, animal studies have suggested that surgery itself had no effect on fear memories in PTSD (30). Third, the population in this study was relatively narrow due to the use of strict inclusion and exclusion criteria. Patients with psychiatric or neurological disorders, such as depression or insomnia, were excluded to decrease the likelihood that the disease itself would interfere with the evaluation of PTSD (31, 32). Four, some studies suggested that PTSD was bound up with chronic pain (33). The lack of data on chronic postsurgical pain may be a limitation in this research. Finally, this study evaluated emergency department trauma patients after only 1 month, and a systematic review of traumatic brain injury showed changes in the prevalence of PTSD after 3, 6, 12, and 24 months (34). Our follow-up studies will extend the duration of follow-up.

In conclusion, early use of propofol general anesthesia in emergency surgery for trauma patients has a certain risk of PTSD. Compared with propofol anesthesia, sevoflurane anesthesia may have certain value in reducing postoperative PTSD in trauma patients undergoing early emergency surgery. Therefore, anesthesia should be carefully selected for patients with trauma requiring emergency surgery as soon as possible, and further research on this issue is needed in the future.

## Data Availability Statement

The original contributions presented in the study are included in the article/supplementary material, further inquiries can be directed to the corresponding authors.

## Ethics Statement

The studies involving human participants were reviewed and approved by the Ethics Committee of Suzhou Xiangcheng People's Hospital. The patients/participants provided their written informed consent to participate in this study.

## Author Contributions

JZ formulated the design of the studies. JZ, YL, and LF performed the experiments and analysis of the studies and drafted the manuscript. DH, CG, SH, RW, LW, RY, and BL performed the experiments and collected data. YY and YZ conceived the study, completed its design and coordination, and secured funding for the project. All authors contributed to the article and approved the submitted version.

## Funding

This study was supported by the Research and Training Program for the Clinical Backbone in Xuzhou (2020GG014) and Science and Technology Development Plan Project of Suzhou (SYSD2019062).

## Conflict of Interest

The authors declare that the research was conducted in the absence of any commercial or financial relationships that could be construed as a potential conflict of interest.

## Publisher's Note

All claims expressed in this article are solely those of the authors and do not necessarily represent those of their affiliated organizations, or those of the publisher, the editors and the reviewers. Any product that may be evaluated in this article, or claim that may be made by its manufacturer, is not guaranteed or endorsed by the publisher.
